# Paediatric critical COVID-19 and mortality in a multinational prospective cohort

**DOI:** 10.1016/j.lana.2022.100272

**Published:** 2022-05-17

**Authors:** Sebastian Gonzalez-Dambrauskas, Pablo Vasquez-Hoyos, Anna Camporesi, Edwin Mauricio Cantillano, Samantha Dallefeld, Jesus Dominguez-Rojas, Conall Francoeur, Anar Gurbanov, Liliana Mazzillo-Vega, Steven L. Shein, Adriana Yock-Corrales, Todd Karsies, Critical Coronavirus and Kids Epidemiological (CAKE) Study Investigators, Ryan Nofziger, Shashikanth Ambati, Tanil Kendirli, Ronald Sanders, Lee Polikoff, Siobhan Whelan, Anna Camporesi, Conall Francoeur, Francisca Castro, Claudia Beltrán, Rosalba Pardo, Gonzalo Vega, Mauricio Yunge, Lorena Acevedo, Ivan Jose Ardila, Diego Aranguiz, Samantha Dallefeld, MarthaI Alvarez-Olmos, Jaime Fernandez-Sarmiento, Arieth Figueroa-Vargas, Maribel Valencia-Benavides, Juan David Roa, Rubén Lasso-Palomino, Alessia Franceschi, Carina Venthur, Sebastian Gonzalez-Dambrauskas, Camila Ampuero, Jhovana E. Paco-Barral, Jaime Tasayco-Muñoz, Jesús Domínguez-Rojas, Francisca Rafael-Patricio, Solana Pellegrini, Marcela Zuazaga, Silvana Brusca, Marisa Viera, Vladmir Ivan Aguilera-Avendano, F. Alejandro, L. Martínez, E. Thelma, M. Terán, Mariela Coronado-Lujan, Fabiola Castro-Mancilla, Franco Diaz-Rubio, Karina Cinquegrana, Alicia Sandoval, Andrea Gonzalez, Marta Zamora, Yurika Lopez-Alarcon, María Slöcker-Barrio, Javier Urbano-Villaescusa, Humberto Camacho, Liliana Mazzillo, Beatriz Giraldo, Pitas Suarez, Miguel Cespedes-Lesczinsky, Jorge Omar Castillo, Juan Pablo Fabris, Carolina Paladino, Silvia Sanabria, Erika Urena-Chavarría, Adriana Yock-Corrales, Gaudi Quispe, Manuel Munaico-Abanto, Miriam Colombo, Ana Carola Blanco, Byron Enrique Pineres-Olave, Ricardo Carvajal-Veas, Patricia Correa, Ricardo Garcia-De-Jesus, Arani Ferre, Pietro Pietroboni, Edwin Mauricio-Cantillano, Linda Banegas-Pineda, Nils Casson-Rodriguez, Agustin Cavagnaro, Adriana Wegner, Eliana Zemanate, Emilce Beltran-Zuñiga, Maria Alejandra Suarez, Deyanira Quiñonez, Leonardo Valero, Alejandra Repetur, Pablo Castellani, Adriana Bordogna, Alfredo De-la-Hoz-Pastor, Evelyn Obando-Belalcazar, Andrew Prout, Roberto Jabornisky, Andy Wen, Bria Coates, Christopher Watson, Elizabeth Mack, Jahee Hong, Todd Karsies, Steven Pon, Heda Dapul, Steven Shein, Murat Kangin, Pablo Vasquez-Hoyos, Shira Gertz, Laurence Ducharme-Crevier, Ilana Harwayne-Gidansky, Marisol Fonseca-Flores, Juan Carlos Nunez-Enriquez, Armando Leon-Villanueva, Ledys Maria, Teddy Muisyo, Michael Spaeder

**Affiliations:** a Red Colaborativa Pediátrica de Latinoamérica (LARed Network) and Cuidados Intensivos Pediátricos Especializados (CIPe) Casa de Galicia, Montevideo, Uruguay; b Red Colaborativa Pediátrica de Latinoamérica (LARed Network), Universidad Nacional de Colombia and Sociedad de Cirugía Hospital de San José, FUCS, Bogota, Colombia; c Division of Pediatric Anesthesia and ICU, Department of Pediatrics. Children's Hospital Vittore Buzzi, Milan, Italy; d UCIP. Hospital Regional del Norte, Instituto Hondureño de Seguridad Social, San Pedro Sula, Honduras; e Pediatric Critical Care Medicine, Dell Children's Medical Center of Central Texas, Austin, TX, USA; f Departamento Pediatría Universidad Nacional Federico Villarreal. UCIP Hospital de Emergencia Villa El Salvador, Lima, Perú; g Department of Pediatrics, Division of Pediatric Critical Care, CHU de Québec – Université Laval Research Center, Québec, Québec, Canada; h Department of Pediatric Critical Care Medicine, Ankara University Faculty of Medicine, Ankara, Turkey; i Pediatric Critical Care Medicine, Hospital infantil Los Ángeles, Pasto, Colombia; j Division of Pediatric Critical Care Medicine, Rainbow Babies and Children's Hospital, Case Western Reserve University, Cleveland, Ohio, USA; k Emergency Department. Hospital Nacional de Niños “Dr. Carlos Sáenz Herrera”, CCSS. San José, Costa Rica; l Division of Pediatric Critical Care, Nationwide Children's Hospital, Columbus, Ohio, USA

**Keywords:** Paediatric intensive care unit, COVID-19, Outcomes, Mortality, Epidemiology

## Abstract

**Background:**

To understand critical paediatric coronavirus disease 2019 (COVID-19) and evaluate factors associated with mortality in children from high and low-middle income countries.

**Methods:**

Prospective, observational study of critically ill children hospitalised for COVID-19 in 18 countries throughout North America, Latin America, and Europe between April 1 and December 31, 2020. Associations with mortality were evaluated using logistic regression.

**Findings:**

557 patients (median age, 8 years; 24% <2 years) were enrolled from 55 sites (63% Latin American). Half had comorbidities. Invasive (41%) or non-invasive (20%) ventilation and vasopressors (56%) were the most common support modalities. Hospital mortality was 10% and higher in children <2 years old (15%; odds ratio 1·94, 95%CI 1·08-3·49). Most who died had pulmonary disease. When adjusted for age, sex, region, and illness severity, mortality-associated factors included cardiac (aOR 2·89; 95%CI 1·2-6·94) or pulmonary comorbidities (aOR 4·43; 95%CI 1·70-11·5), admission hypoxemia (aOR 2·44; 95%CI 1·30-4·57), and lower respiratory symptoms (aOR 2·96; 95%CI 1·57-5·59). MIS-C (aOR 0·25; 95%CI 0·1-0·61) and receiving methylprednisolone (aOR 0·5; 95%CI 0·25-0·99), IVIG (aOR 0·32; 95%CI 0·16-0·62), or anticoagulation (aOR 0·49; 95%CI 0·25-0·95) were associated with lower mortality although these associations might be limited to children >2 years old.

**Interpretation:**

We identified factors associated with COVID-19 mortality in critically ill children from both high and low-middle income countries, including higher mortality with younger age and COVID-related pulmonary disease but lower mortality in MIS-C. Further research is needed on optimal treatments for younger children and respiratory failure in paediatric COVID-19.

**Funding:**

None.

**Research in context**

**Evidence before this study**

We searched MEDLINE on February 2, 2022, through PubMed to identify publications reporting on critical COVID-19 in children. The search terms used were "COVID-19 AND (Critical Illness OR Critical Care)" with an age limit from birth to18-year-old. No additional limits were set. This search yielded 1,293 results. We reviewed case series, observational studies, and systematic reviews with at least five cases total and with at least one critical or paediatric intensive care unit (PICU) case and selected 83 manuscripts. Most manuscripts were from 2020, represented small observational studies mainly in high income countries (HIC) and used different definitions for critical COVID-19. The best estimates for paediatric COVID-19 mortality comparing population-based data between HIC and low- and middle-income countries settings (LMIC) came from Kitano *et a*l., which found that LMICs accounted the majority (90%) of paediatric COVID-19 deaths and had higher mortality rates (incidence 2·77 cases per 1,000,000 children versus 1·32 in HICs). However, studies of critical paediatric COVID-19 have primarily involved HIC cohorts and evaluated risk factors associated with PICU admission and ICU disease course in resource-rich settings. Furthermore, most paediatric ICU studies include all patients admitted to the ICU regardless of whether they are actually critically ill. LMICs are still largely underrepresented although there are data signalling a different impact on mortality according to country of origin.

**Added value of this study**

Our study collected data from a large multinational cohort from both HICs and LMICs across Europe, North America, and Latin America during the first year of the pandemic. Latin America, in particular, is a region that has been largely ignored in paediatric critical COVID-19 research but comprised over 60% of our broad international cohort. We included only those who were critically ill and either received ICU-level respiratory, cardiovascular, or renal support or were in the ICU and receiving high levels of oxygen support other than positive pressure ventilation. We identified a much higher overall mortality than previously reported in critical paediatric COVID-19, especially among children <2 years old. We also found differences in disease phenotypes and early treatments that were associated with mortality. A respiratory phenotype, more common in younger children, was associated with higher mortality, while an inflammatory or cardiovascular phenotype, such as multisystem inflammatory syndrome (MIS-C), was more common in older children and associated with lower mortality.

**Implications of all the available evidence**

The higher mortality, especially among younger children, has implications for public health and vaccination strategies in LMICs and should prompt continued paediatric-specific research examining risks for mortality (with special focus on regional and contextual causes of variability of clinical care and outcomes) and determining the best treatments for critical paediatric COVID-19.

Alt-text: Unlabelled box

## Introduction

Evidence from the first year of the coronavirus disease 2019 (COVID-19) pandemic indicated that affected children typically develop mild, often asymptomatic, disease and are less likely to require hospitalization than adults.^1^^,^^2^ However, when children do require hospitalization, up to one-third require ICU admission.^2^^,^^3^ Few studies focus on paediatric critical COVID-19, and most larger studies are from high-income countries (HICs).^4^, ^5^, ^6^, ^7^ Studies examining paediatric ICU (PICU) admissions from low-middle-income countries (LMICs) are scarce and generally focus on multisystem inflammatory syndrome in children (MIS-C).^8^, ^9^, ^10^

In addition to less severe presentation, children have lower mortality compared to adults, even when hospitalized, although mortality appears to be much higher in LMICs.^1^^,^^2^^,^^11^ A systematic review found that over 90% of paediatric COVID-19 deaths were from LMICs and that mortality risk was highest in children under 1 year-old.^12^ Mortality differences can be partly explained by resource limitations, but the reasons for age-related differences are unclear. Potential explanations include developmental immune differences, lack of prior exposure to other coronaviruses, and less frequent development of MIS-C, which might have lower mortality compared to COVID-19-related acute respiratory disease.^13^^,^^14^ This mortality risk is further complicated by the fact that young children are currently unable to receive SARS-CoV2 vaccination because of lack of regulatory approval, the perception that young children are unlikely to have severe infections, and public health strategies that prioritize adult vaccination.

To understand the epidemiology and outcomes of critical paediatric COVID-19 in both HICs and LMICs, we designed the Critical Coronavirus And Kids Epidemiology (CAKE) study, which includes PICUs from North, Central, and South America plus Europe. We previously reported a case series with preliminary insights into paediatric critical COVID-19 across these regions.^15^ The primary objective of this manuscript was to identify risk factors associated with mortality in critical paediatric COVID-19. The secondary objective was to describe the epidemiology, clinical findings, and hospital treatments of critical COVID-19 in children from both HICs and LMICs with a focus on early treatments since these may represent modifiable factors. Due to suspected differences in presentation and outcomes based on age (<2 years old vs ≥2 years old) in our experience with other respiratory viruses (i.e., respiratory syncytial virus) and suspected differences between MIS-C and other forms of paediatric COVID-19, we explored outcomes in subgroups based on age and MIS-C diagnosis.

## Methods

### Study design and setting

CAKE is a prospective, observational cohort study examining the epidemiology and outcomes of children hospitalized for severe or critical COVID-19. We followed STROBE guidelines when reporting.

### Participants

We enrolled patients <19 years old hospitalized between April 1 and December 31, 2020, with severe or critical confirmed COVID-19 and/or MIS-C. Patients were enrolled if they met the following criteria:

• Laboratory confirmation of current or prior infection by SARSCoV2 using polymerase chain reaction, antigen, or serologic testing depending on laboratory availability.
• COVID-19 related illness as the primary reason for hospitalization

And either

• Severe or critical COVID-19 based on our prior definition ^15^

Or

• MIS-C requiring ICU admission, even if not meeting our definition of severe/critical COVID-19.

Children admitted to an ICU without either meeting our criteria for severe/critical COVID-19 or having MIS-C were not eligible for inclusion.

### Definitions

Critical COVID-19 was defined as having a positive SARS-CoV2 test and requiring at least one of the following therapies: ICU-level respiratory support including high flow nasal cannula (HFNC), non-invasive ventilation (NIV), or invasive mechanical ventilation (IMV); intravenous vasopressor or inotrope support; or continuous renal replacement therapy (RRT). Severe COVID-19 included patients with a positive SARS-CoV2 test who did not meet critical COVID-19 criteria but received oxygen support via mask or nasal cannula exceeding the paediatric acute respiratory distress syndrome (ARDS) “at-risk” criteria.^16^ MIS-C cases were defined using the Centers for Disease Control and Prevention (CDC) criteria.^17^ Because shortness of breath and chest pain can both be associated with cardiovascular and respiratory processes, we categorized these symptoms as “non-specific”; other nonspecific symptoms include myalgias, arthralgias, and lymphadenopathy.

### Data collection, variables, and outcome measures

Prospective de-identified data were collected using a modification of the case report form developed by the International Severe Acute Respiratory and emerging Infection Consortium (ISARIC). We used REDCap (Research Electronic Data Capture, Vanderbilt University) online database hosted by Nationwide Children's Hospital (Columbus, Ohio, USA). Collected data included demographics, pre-hospitalization comorbidities, presenting symptoms, exposures, microbiology, ICU and hospital treatments (including organ support and medications), treatment durations, hospital and PICU length of stay (LOS), and in-hospital mortality. Standardized definitions for comorbidities and diagnoses were not used, but these data were obtained from the medical records at each site based on clinician documentation. Data on illness progression and severity were collected daily from hospital day 1 until day 14, death, or hospital discharge. Illness severity was estimated using the Pediatric Risk of Mortality III (PRISM III) and Pediatric Logistic Organ Dysfunction 2 (PELOD-2) scores.^18^^,^^19^ Diagnoses and complications were based on clinician documentation. The primary outcome was in-hospital mortality. Missing data were completed after the enrolment period through record review by site investigators. Patients with incomplete data related to the initial presentation, day 1 treatments, or outcomes were excluded from the final analysis.

### Data analysis

Categorical variables were described as frequencies and percentages, and continuous variables expressed as medians and interquartile ranges (IQRs). We used chi-square test to analyse categorical variables and Wilcoxon rank-sum test for continuous variables. We performed univariate and multivariate logistic regression to examine associations with mortality; results from regression models are reported as odds ratios (unadjusted or adjusted) and 95% confidence intervals (CI). Statistical significance was designated as p value ˂0·05 (2-sided) or a 95% confidence interval that does not include 1. For our multivariable model, we included variables that were statistically significant in univariate logistic regression as well as those associated with mortality in other studies. Multivariable models were adjusted for age (as a continuous variable), sex, region (Latin America vs. non-Latin America), and PRISM III, which were selected *a priori* due to suspicion that they may be associated with outcomes. We also performed stratified logistic regression to evaluate differences between children <2 years and older children as well as children with and without MIS-C. In addition, we evaluated the categorical variable age <2 years old for interactions with covariates in logistic regression. We were unable to perform any sample size estimates because data about paediatric critical COVID-19 and mortality did not exist at the time this study began. Statistical analysis was performed using JMP 15 Pro for Mac (SAS Institute, Cary, North Carolina). Because we performed multiple comparisons between variables and mortality, results of this study should be considered exploratory.

### Ethics

The overall study was approved by the institutional review board of Nationwide Children's Hospital (STUDY00000966) as well as at each participating site with a waiver of need for informed consent.

### Role of the funding source

This study was unfunded.

## Results

A total of 84 sites screened patients, and 590 patients were enrolled from 55 different sites. After excluding 33 patients due to incomplete data from presentation, day 1 treatments, or outcomes, 557 patients from the 55 sites were analysed. Of these, 433 met the criteria for critical COVID-19, 76 met our definition for severe COVID-19, and 48 were in the PICU solely with MIS-C.

The median age was 8 (IQR 2, 12·4) years with 24% under 2 years old and 61% male (Table 1). Only 43% had a known COVID-19 exposure. There were 352 (63%) from Latin America (Supplemental Table 1). Comorbidities were present in half of the patients (Table 1).

**Table 1:** Demographics, comorbidities, and clinical presentation of children with critical COVID-19.

Variable	All (*n*=557)^a^	Survived (*n*=501)^a^	Died (*n*=56)^a^	*p*-value
**Demographic**s				
Age, years	8 (2, 12·4)	8 (2·06, 12·4)	4 (1, 12·7)	0·048
Male Sex	338 (61%)	305 (61%)	33 (59%)	0·777
**Comorbidities**				
Cardiac	43 (7·7%)	33 (6·6%)	10 (18%)	0·003
Pulmonary (not asthma)	32 (5·7%)	24 (4·8%)	8 (14%)	0·004
Asthma	44 (7·9%)	39 (7·8%)	5 (8·9%)	0·763
Renal	22 (4%)	19 (3·8%)	3 (5·4%)	0·569
Liver	9 (1·6%)	5 (1%)	4 (7·1%)	<0·001
Neurologic	58 (10%)	49 (9·8%)	9 (16%)	0·144
Malignancy	20 (3·6%)	13 (2·6%)	7 (13%)	<0·001
Hematologic	18 (3·2%)	13 (2·6%)	5 (8·9%)	0·011
Obesity	74 (13%)	70 (14%)	4 (7·1%)	0·153
Diabetes	11 (2%)	10 (2%)	1 (1·8%)	0·915
Malnutrition	36 (6·4%)	26 (5·2%)	10 (18%)	<0·001
>1 Comorbidity	119 (21%)	93 (19%)	26 (46%)	<0·001
No Comorbidities	272 (49%)	257 (51%)	15 (27%)	<0·001
**Clinical Presentation**				
Known COVID Exposure	240/555 (43%)	217/499 (43%)	23 (41%)	0·729
Day of Illness at Admission	4 (2, 6)	4 (2, 6)	3 (1·2, 7)	0·746
ICU During Hospitalization	540/555 (97%)	486/499 (97%)	54 (96%)	0·672
Ward Admission Pre-ICU	249/527 (47%)	220/475 (46%)	29/52 (56%)	0·195
Fever (>38·3°C) at Admission	112/542 (21%)	106/487 (22%)	6/55 (11%)	0·059
Admission O_2_ Saturation <94%	116/533 (22%)	95/478 (20%)	21/55 (38%)	0·002
Dehydration at Admission	87/555 (16%)	77/499 (15%)	10 (18%)	0·636
Delayed Admission Capillary Refill	141/370 (38%)	116/327 (35%)	25/43 (58%)	0·004
PRISM III at ICU Admission	5 (2, 10)	5 (2, 10)	10·5 (4·25, 13·75)	<0·001
PELOD 2 at ICU Admission	2 (0, 6)	2 (0, 5·5)	5 (2, 9)	<0·001
**Presenting Symptoms**				
Lower Respiratory	262 (47%)	221 (44%)	41 (73%)	<0·001
Upper Respiratory	165 (30%)	154 (31%)	11 (20%)	0·085
Systemic	469 (84%)	423 (84%)	46 (82%)	0·656
Gastrointestinal	327 (59%)	304 (61%)	23 (41%)	0·005
Neurologic	214 (38%)	190 (38%)	24 (43%)	0·472
Mucocutaneous	208 (37%)	198 (40%)	10 (18%)	0·002
Non-Specific	299 (54%)	263 (53%)	36 (64%)	0·093

Data are n (%) or median (IQR).

Abbreviations: PRISM III = Pediatric Risk of Mortality III; PELOD-2 = Pediatric Logistic Organ Dysfunction 2.

a Sample size unless otherwise noted. Lower sample size due to unavailable data.

Presenting symptoms and clinical findings are noted in Table 1 and Supplemental Table 2. Nearly half were admitted to the ward prior to ICU. The most common ICU-level support modalities included vasopressors (56% of patients), IMV (41% of patients), and NIV (20% of patients) (Table 2). Vasopressors/inotropes were used for a median of 3 days (IQR 2, 5), and the median IMV duration was 5 days (IQR 2·9, 9). The median ICU LOS for survivors was 5 days (IQR 3, 8·8), and hospital LOS was 10 days (IQR 6, 15) and the overall hospital mortality rate was 10·1%.

**Table 2:** Organ support and medications and associations with mortality. Odds ratios from univariate logistic regression.

Treatment	Mortality		Logistic Regression	
	With Treatment	Without Treatment	Odds Ratio	95% CI
**Day 1 Support**				
HFNC	3/73 (4·1%)	53/474 (11%)	0·34	0·10, 1·12
NIV	3/54 (5·6%)	52/490 (11%)	0·50	0·15, 1·64
IMV	28/134 (21%)	28/412 (6·8%)	**3·62**	2·06, 6·38
Any PPV	31/178 (17%)	25/379 (6·6%)	**2·99**	1·70, 5·23
Vasopressors	29/196 (15%)	27/348 (7·8%)	**2·06**	1·18, 3·60
NMB	9/43 (21%)	46/501 (9·2%)	**2·62**	1·18, 5·80
Prone Position	4/10 (40%)	50/533 (9·4%)	**6·44**	1·76, 23·6
RRT	4/13 (31%)	52/529 (9·8%)	**4·08**	1·21, 13·7
**Hospital Support**				
NIV	9/109 (8·3%)	47/447 (11%)	0·77	0·36, 2·75
IMV	51/228 (22%)	5/329 (1·5%)	**18·7**	7·32, 47·6
Any PPV	52/289 (18%)	4/268 (1·5%)	**14·5**	5·16, 40·6
Vasopressors	51/312 (16%)	5/244 (2·1%)	**9·34**	3·67, 23·8
Prone	16/42 (38%)	40/514 (7·8%)	**7·29**	3·62, 14·7
RRT	14/27 (52%)	42/529 (7·9%)	**12·5**	5·51, 28·3
**Hospital Medications**				
Corticosteroids	34/391 (8·7%)	22/165 (13%)	0·62	0·35, 1·1
Dexamethasone	17/157 (10·8%)	39/400 (9·8%)	1·12	0·62, 2·06
Methylprednisolone	12/220 (5·5%)	44/337 (13%)	**0·38**	0·20, 0·75
IVIG	13/262 (5%)	43/295 (15%)	**0·31**	0·16, 0·58
IVIG and methylprednisolone	5/154 (3·3%)	51/403 (13%)	**0·23**	0·09, 0·59
IVIG or methylprednisolone	20/327 (6·1%)	36/230 (16%)	**0·35**	0·20, 0·62
Any Anticoagulation	14/244 (5·7%)	42/313 (13%)	**0·39**	0·21, 0·74
Prophylactic Anticoagulation	11/195 (5·6%)	45/362 (13%)	**0·42**	0·21, 0·83
Antiplatelet	1/101 (1%)	55/456 (12%)	**0·07**	0·01, 0·53
Exchange Transfusion	2/8 (25%)	54/549 (9·8%)	3·06	0·60, 15·5
Convalescent Plasma	2/12 (17%)	54/545 (9·9%)	1·82	0·39, 8·51
Anakinra	1/23 (4·4%)	55/534 (10%)	0·40	0·05, 2·99
Tocilizumab	2/10 (20%)	54/547 (9·9%)	2·28	0·47, 11·0
Hydroxychloroquine/Chloroquine	4/21 (19%)	52/536 (9·7%)	2·19	0·71, 6·75

Statistically significant results in **bold**.

### Factors related with mortality

Overall, mortality was higher in younger children [15% (20/134) in those <2 years old vs 8% (35/422) in those ≥2] and in those without MIS-C [13% (49/367) vs 3·2% (6/188) in those with MIS-C] (*Supplemental Figure* 1). The largest age-related differences were seen in those without MIS-C (*Supplemental Figure* 2). Notably, cardiac arrest was associated with an 85% mortality, while four of five patients treated with extracorporeal membrane oxygenation (ECMO) survived. Children who died more commonly had comorbid conditions, lower respiratory symptoms, documented hypoxemia, or delayed capillary refill at admission (Table 1; *Supplemental Table* 3). They also had more severe illness at ICU admission as measured by PRISM III and PELOD 2 (Table 1). Children who survived more commonly had gastrointestinal or mucocutaneous symptoms and a documented fever at admission. Although labs were frequently obtained on day 1, this was not systematically done, and most were not associated with mortality. Non-survivors had a lower pH, higher blood urea nitrogen (BUN), and higher lactate on day 1, while survivors had lower sodium and higher C-reactive protein and procalcitonin (*Supplemental Table* 4).

**Figure 1:**
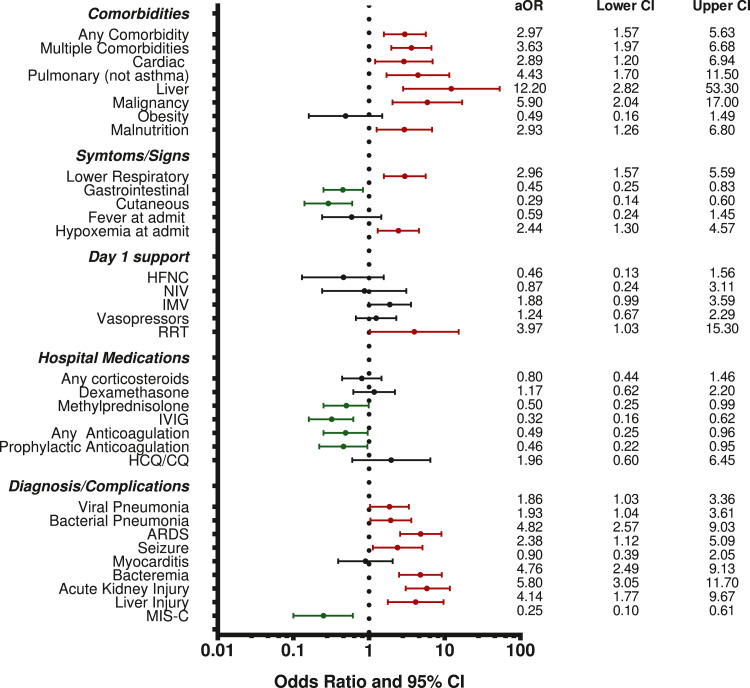
Multivariable Associations with Mortality. Odds ratio for each variable adjusted for sex, age<2, region, and admission PRISM III. Abbreviations: PRISM III= Pediatric Risk of Mortality III; HFNC = high flow nasal cannula; NIV = noninvasive ventilation; IMV = invasive mechanical ventilation; RRT = renal replacement therapy; IVIG = intravenous immune globulin; HCQ/CQ = hydroxychloroquine or chloroquine; ARDS = acute respiratory distress syndrome; MIS-C = multisystem inflammatory syndrome in children.

In univariate logistic regression, multiple treatments were associated with mortality (Table 2). Requiring IMV, vasopressors, prone positioning, neuromuscular blockade, and renal replacement therapy were all associated with increased mortality. Having a pulmonary or neurologic diagnosis plus developing renal or hepatic dysfunction was also associated with higher mortality while having MIS-C was associated with lower mortality (*Supplemental Table* 5). When adjusted for age, sex, region (Latin America vs non-Latin America), and initial PRISM III score, having comorbidities (particularly cardiac, pulmonary, liver disease, malignancy, malnutrition), presenting with lower respiratory symptoms, having admission hypoxemia, receiving RRT on day 1, and a diagnosis of pneumonia (viral or bacterial), ARDS, seizures, bacteraemia, AKI, and acute hepatic injury remained associated with higher mortality (Figure 1). Presenting with gastrointestinal or mucocutaneous symptoms, treatment with methylprednisolone and/or IVIG or anticoagulation, and having a diagnosis of MIS-C were associated with lower mortality when adjusting for age, sex, region, and initial PRISM III. Dexamethasone was not associated with improved mortality.

### MIS-C subgroup analyses

**Figure 2:**
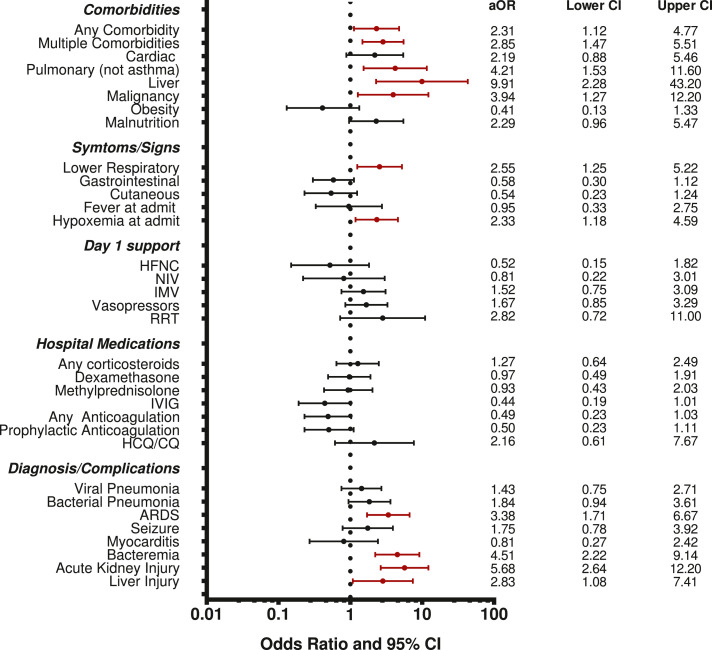
Multivariable Associations with Mortality in the subset of children without MIS-C. Odds ratio for each variable adjusted for sex, age<2, region, and admission PRISM III. Abbreviations: PRISM III= Pediatric Risk of Mortality III; HFNC = high flow nasal cannula; NIV = noninvasive ventilation; IMV = invasive mechanical ventilation; RRT = renal replacement therapy; IVIG = intravenous immune globulin; HCQ/CQ = hydroxychloroquine or chloroquine; ARDS = acute respiratory distress syndrome; MIS-C = multisystem inflammatory syndrome in children.

Given the differences in the disease process between MIS-C and other forms of acute COVID-19 plus the extremely low MIS-C mortality, we evaluated the factors associated with mortality for the other forms of acute COVID-19 (non-MIS-C subgroup) (Table 3). In adjusted analysis, the presence of any or multiple comorbidities (and specifically pulmonary, liver, and malignancy comorbidities); lower respiratory symptoms; hypoxemia at admission; and having a diagnosis of ARDS, bacteraemia, AKI, or hepatic injury remained were associated with higher mortality (Figure 2). No specific medications were associated with mortality. We did not carry out subgroup analysis of MIS-C mortality because there were only 6 total deaths with MIS-C (3·2%). Half of MIS-C deaths occurred in children <2 years even though they comprised only 10% of all MIS-C patients.

**Table 3:** Subgroup analysis of treatments associated with mortality for those without a diagnosis of MIS-C.

Treatment	Mortality		Logistic Regression	
	With Treatment	Without Treatment	Odds Ratio	95% CI
**Patients without MIS-C**^a^				
Day 1 HFNC	3/57 (5·3%)	47/308 (15%)	0·31	0·09, 1·03
Day 1 NIV	3/42 (7·1%)	46/321 (14%	0·46	0·14, 1·55
Day 1 IMV	26/108 (24%)	24/256 (9·4%)	**3·07**	1·67, 5·64
Day 1 Vasopressors	27/111 (24%)	23/251 (9·2%)	**3·19**	1·73, 5·86
Day 1 Neuromuscular Blockade	9/37 (24%)	40/325 (12·3%)	**2·29**	1·01, 5·20
Day 1 Prone Position	4/10 (40%)	44/351 (13%)	**4·65**	1·26, 17·1
Day 1 Renal Replacement	4/13 (31%)	46/348 (13%)	2·92	0·86, 9·86
*Hospital Medications*				
Corticosteroids	31/234 (13%)	19/133 (14%)	0·92	0·50, 1·70
Dexamethasone	16/135 (12%)	34/233 (15%)	0·79	0·42, 1·49
Methylprednisolone	10/90 (11%)	40/278 (14%)	0·74	0·36, 1·56
IVIG	8/96 (8·3%)	42/272 (15%)	0·50	0·22, 1·10
IVIG and methylprednisolone	3/34 (8·8%)	47/334 (14%)	0·59	0·17, 2·01
IVIG or methylprednisolone	15/151 (9·9%)	35/217 (16%)	0·57	0·30, 1·09
Any Anticoagulation	12/141 (8·5%)	38/227 (17%)	**0·46**	0·23, 0·92
Prophylactic Anticoagulation	10/117 (8·6)	40/251 (16%)	0·49	0·24, 1·02
Convalescent Plasma	2/11 (18%)	48/357 (13%)	1·43	0·30, 6·82
Hydroxychloroquine/Chloroquine	4/15 (27%)	46/353 (13%)	2·43	0·74, 7·94

Statistically significant results in **bold**.

a Excluded antiplatelet medications and anakinra in the non-MISC subgroup due to low numbers receiving the treatment.

### Age subgroup analyses

Because we also suspected that clinical factors and treatments may impact mortality in younger children differently than older children, we explored associations with mortality using univariate logistic regression stratified by age <2 and ≥2 years old (Table 4; *Supplemental Table* 6). Although most factors significantly associated with mortality in the entire cohort had similar associations in stratified analysis, a diagnosis of MIS-C or symptoms typically found in MIS-C (gastrointestinal or cutaneous) plus treatments frequently used for MIS-C (methylprednisolone, IVIG, prophylactic anticoagulation) were associated with lower mortality only in older children. Having viral pneumonia or acute liver injury was associated with higher mortality only in younger children. A statistically significant interaction with age was seen for treatment with IVIG or methylprednisolone plus a diagnosis of viral pneumonia, acute liver injury, and MIS-C. Of note, 40% of children >2 years old had MIS-C while only 15% of those <2 years old had MIS-C. In a *post hoc* analysis statistically significant mortality differences between older and younger children were seen only for those with MIS-C (15% versus 1.8%; p=0·002) and not for those without MIS-C (15% versus 13%; p=0·56).

**Table 4:** Univariate associations with mortality plus mortality rate for each variable stratified by age. Includes factors significantly associated with mortality from multivariable analysis for the entire cohort.

	<2 years old			≥2 years old		
Variable	OR	95% CI	Mortality	OR	95% CI	Mortality
Admission O_2_ Saturation <94%	2·36	0·87, 6·44	25%	2·48	1·19, 5·16	15%
Pulmonary Comorbidity	1·70	0·33, 8·83	22%	4·35	1·60, 11·9	26%
Malnutrition	3·47	1·04, 11·5	33%	3·74	1·28, 10·9	24%
Lower Respiratory Symptoms	5·47	1·52, 19·7	23%	2·75	1·34, 5·66	13%
Gastrointestinal Symptoms	0·85	0·29, 2·54	14%	0·42	0·21, 0·84	6·2%
Mucocutaneous Symptoms	0·56	0·12, 2·60	9·5%	0·33	0·15, 0·75	4·3%
Day 1 Invasive Ventilation	3·37	1·27, 8·97	28%	3·53	1·75, 7·12	18%
Prophylactic Anticoagulation	0·94	0·25, 3·55	14%	0·38	0·17, 0·86	4·6%
**IVIG**^a^	**1·15**	**0·40, 3·25**	**16%**	**0·19**	**0·08, 0·44**	**3·1%**
Dexamethasone^b^	0·93	0·31, 2·79	14%	1·26	0·61, 2·61	9·8%
**Methylprednisolone**^a^	**1·32**	**0·43, 4·00**	**18%**	**0·26**	**0·11, 0·62**	**3·7%**
**Viral Pneumonia**^a^	**4·60**	**1·56, 13·5**	**25%**	**1·24**	**0·60, 2·56**	**9·7%**
ARDS	7·17	2·52, 20·4	33%	6·51	3·13, 13·5	21%
Bacteraemia	5·93	2·11, 16·6	36%	6·41	2·90, 14·2	30%
Acute Kidney Injury	14·5	4·10, 51·6	62%	4·45	2·18, 9·08	21%
**Acute Liver Injury**^a^	**14·0**	**2·37, 82·7**	**67%**	**2·22**	**0·91, 5·40**	**16%**
**MIS-C**^a^	**1·01**	**0·26, 3·76**	**15%**	**0·12**	**0·04, 0·40**	**1·8%**

IVIG = intravenous immune globulin; ARDS = acute respiratory distress syndrome.

MIS-C = multisystem inflammatory syndrome in children.

a Variables with a statistically significant interaction with age in logistic regression analysis noted in **bold**.

b Analysis of dexamethasone was included despite not having a significant association with mortality in adjusted analysis due to adult data and the wide recommendation for its use in children with COVID-19.

## Discussion

Our study of critical paediatric COVID-19 from HICs and LMICs observed higher mortality than previously reported in large PICU studies and identified several factors associated with mortality.^4^^,^^6^^,^^7^ Having a primarily respiratory illness was associated with higher mortality, while developing MIS-C or receiving treatments typically used for MIS-C was associated with lower mortality. Comorbid medical conditions were associated with higher mortality, both overall and in the subset of patients without MIS-C. Dexamethasone was not associated with improved mortality in any illness presentation or age group.

The overall high PICU mortality observed in our study contrasts with most previous paediatric reports. This could be explained by the fact that most large PICU studies have studied children from HICs where mortality is low and primarily occurred in older children with comorbidities 4, 5, 6, 7^,^^20^^,^^21^. Our findings parallel the paediatric COVID-19 mortality differences between HICs and LMICs described by Kitano and suggest that lower PICU mortality in HICs might not translate to LMICs.^12^ Besides some Brazilian cohorts with a socioeconomic profile similar to HIC populations, most studies from LMICs demonstrate higher mortality, with younger age identified as a mortality risk 8, 9, 10^,^^22^, ^23^, ^24^, ^25^. We achieved significant representation from Latin America, a region largely ignored in prior paediatric studies. Our relatively large sample size, broad geographic representation, and high illness severity allowed us to evaluate mortality in ways not feasible in prior studies of critical paediatric COVID-19. Also, our higher respiratory failure and lower MIS-C rates plus restrictive eligibility criteria likely resulted in a sicker cohort than other large studies that included children who were not critically ill or had lower rates of ARDS, IMV use, and vasopressors use.^4^^,^^6^^,^^7^ Our cohort may then better reflect the full paediatric illness spectrum and severity that may be encountered in critical COVID-19 around the world. Inclusion of PICUs from diverse contexts may better allow generalizability to paediatric critical illness due to COVID-19 at a global scale. In addition, use of an established data tool (the ISARIC report form) and established regional PICU networks such as LARed Network and the Pediatric Acute Lung and Sepsis Investigators (PALISI) network may allow for international comparisons, consistency in data collection, and comparisons with other studies using the same data tools.

Our results also suggest there might be distinct age-related phenotypes in paediatric critical COVID-19. Younger children more commonly had respiratory disease, hypoxemia, and secondary bacterial infections, which all might confer a higher mortality risk. We saw higher IMV use in young children, both compared to older children in our study and to other studies, which might be explained by recommendations early in the pandemic to avoid NIV and intubate early.^4^^,^^6^^,^^7^ Higher IMV use combined with intubation risks may partly explain the lower survival. Of note, we previously described intubation-related cardiac arrest, and here found that over half of young children intubated day 1 also suffered cardiac arrest.^15^ In contrast, older children were more frequently diagnosed with and received treatments for MIS-C. Besides the higher respiratory failure and lower MIS-C rates in younger children, age-related differences included a lack of association between some treatments (IVIG, methylprednisolone, and prophylactic anticoagulation) or diagnoses (MIS-C) and improved mortality in younger children. Some factors that differed (GI or cutaneous symptoms and anticoagulation) did not show statistically significant interactions with age so may not be truly associated with mortality. However, this higher mortality, even when younger children had MIS-C or received treatments associated with better outcomes, is concerning, and research is ongoing to better understand the impact of age and patient factors on outcomes. While the impact of age on outcomes appears dramatic in our data, prior studies have had conflicting findings.^26^^,^^27^ However, these studies evaluated children in HICs and had lower overall mortality. Our observed age-related mortality differences are consistent with data from LMICs, and these regional differences deserve further investigation to understand the causative factors.^12^^,^^23^ Since mortality for those without MIS-C appear similar across age ranges, it seems more likely that age-related mortality differences are due to the higher frequency and better survival rates of MIS-C in children >2 years old rather than worse survival rates for other forms of critical COVID-19 in younger children although further research is needed to explore this association.

While the Recovery trial demonstrated improved outcomes for adults requiring IMV that received dexamethasone, paediatric data are lacking.^28^ We did not find an association between dexamethasone and survival, highlighting the challenge of extrapolating from adult trials to paediatric practice. Several therapies, most frequently used for MIS-C, were associated with lower mortality, including methylprednisolone, IVIG, and prophylactic anticoagulation, although this seems to be limited to older children. Given the association between MIS-C itself and lower mortality, it is possible that these findings may be due to MIS-C rather than the therapies. With limited data supporting many MIS-C and other COVID-19 treatments, it is important to study therapies for children in a prospective and multicentre fashion.^29^, ^30^, ^31^, ^32^

Our findings have several implications, particularly regarding LMICs and age-related outcomes. Current literature evaluating critical paediatric COVID-19 in LMICs or COVID-related respiratory failure is inadequate. There has been more attention on MIS-C, a novel condition associated with very low mortality, rather than the illness phenotypes that we and others have found to have higher mortality.^12^ COVID-related respiratory failure warrants further investigation to determine whether care and outcomes can be improved, particularly in resource-limited settings. These efforts are urgent since vaccine distribution is limited in many LMICs, and younger children (i.e. under 2 years) are unlikely to be vaccinated soon due to lower population-based mortality risk and the unclear balance between vaccine benefit and risk in this age range.^33^ Finally, since some believe that COVID-19 will become a seasonal infection rather than being eradicated, studies are needed to determine the optimal treatments for critical paediatric COVID-19.^34^

Our study does have limitations. As an observational study, we cannot determine causality between treatments and outcomes. Unrecognized factors might be responsible for the higher mortality observed with certain ages, presentations, or treatments. Since we do not have data on when patients developed diagnoses or complications, it is not possible to determine the actual impact these had on mortality. Further, while we had a relatively large sample size and number of deaths compared to other paediatric studies, mortality was still infrequent, limiting our ability to account for confounders. Although we had sites from many countries, we included a minority of PICUs in most countries, which may limit generalizability. Even with this limitation, our study involved a wider range of countries than other studies of critical paediatric COVID-19, providing a perspective on populations underrepresented in prior research. We did not account for regional variation in patients, clinical practice, and outcomes but acknowledge that these likely impacts outcomes and are currently gathering data to study this in our cohort. Since our study ended in December 2020, extrapolations to later pandemic phases with new viral variants, new therapies, and rising paediatric vaccination rates may be challenging. However, the clinical spectrum seen in our cohort seems to be similar to what has been noted with some SARS-CoV2 variants. Since we only included patients who required ICU-level organ support, it may not be appropriate to apply our data to children who are housed in an ICU but not critically ill. Lastly, we do not have data following discharge to capture late mortality or delayed complications such as long COVID. Future studies are needed evaluate long term outcomes after paediatric critical COVID-19.

## Conclusions

This large, prospective, international cohort study of critically ill children with COVID-19 identified mortality-related factors and potential age-related variations in disease phenotypes and mortality. A respiratory phenotype, more common in younger children, was associated with higher mortality. An inflammatory or cardiovascular phenotype (commonly MIS-C) was more common in older children and associated with lower mortality. We identified a higher overall mortality rate than previously reported in critical paediatric COVID-19. The higher mortality, especially among younger children, has implications for public health and vaccination strategies in LMICs and should prompt continued paediatric-specific research examining risks for mortality and determining the best treatments for critical paediatric COVID-19.

## Authors’ Contributions

Drs González-Dambrauskas and Vásquez-Hoyos conceptualized and designed the study, participated in data collection, had full access to all the data in the study and take responsibility for the integrity of the data and the accuracy of the data analysis, helped to draft the initial manuscript, and reviewed and revised the manuscript.

Dr. Karsies conceptualized and designed the study, coordinated, and supervised all aspects of the study, participated in data collection, performed initial data analysis, had full access to all the data in the study and take responsibility for the integrity of the data and the accuracy of the data analysis, helped to draft the initial manuscript, and reviewed and revised the manuscript.

Drs. Camporesi, Cantillano, Dallefeld, Dominguez-Rojas, Francoeur, Gurbanov, Mazzillo Vega, Shein, and Yock-Corrales all participated in data collection, reviewed, and revised the manuscript*,* and critically reviewed the manuscript for important intellectual content.

All authors reviewed the final manuscript as submitted and agree to be accountable for all aspects of the work.

## Funding

This study was unfunded.

## Data sharing

Data collected for the study, including individual deidentified participant data and a data dictionary, will be made available to other reasearcheres after one year from publication upon resonable request. Proposals should be directed to Todd.Karsies@nationwidechildrens.org and to gain access data requestors will need to sign a data access agreement.

## Declaration of interests

No authors have any conflicts of interest to disclose

## Appendix Supplementary materials

Image, application 1

Image, application 2

Image, application 3

Image, application 4

Image, application 5
