# Supplementary material for: Paediatric critical COVID-19 and mortality in a multinational prospective cohort
Source: Lancet Reg Health Am. 2022 May 17;12:100272. doi: 10.1016/j.lana.2022.100272 (PMC9111167; doi:10.1016/j.lana.2022.100272)
Supplement: Supplementary file 1 [file mmc1.docx]

CAKE Study Supplemental Tables and Figures
