# Supplementary material for: Paediatric critical COVID-19 and mortality in a multinational prospective cohort
Source: Lancet Reg Health Am. 2022 May 17;12:100272. doi: 10.1016/j.lana.2022.100272 (PMC9111167; doi:10.1016/j.lana.2022.100272)

Supplemental Data Tables and Figures

Supplemental Table 1: Number of patients and study sites per country **Page 2**

Supplemental Table 2: Comparison of symptoms at presentation **Page 3**

Supplemental Table 3: Association of Comorbidities and Clinical Presentation with Mortality **Page 4**

Supplemental Table 4: Laboratory abnormalities identified on hospital day 1 significantly associated with mortality **Page 5**

Supplemental Table 5 Complete list of hospital diagnoses and complications including odds ratio for mortality of each diagnosis **Page 6**

Supplemental Table 6. Mortality rates for factors significantly associated with mortality in adjusted logistic regression stratified by age **Page 7**

Supplemental Figure 1. Hospital mortality rate stratified by age **Page 8**

Supplemental Figure 2. Hospital mortality stratified by age and MIS-C diagnosis **Page 9**

Supplemental Table 1: Number of patients and study sites per country

Country	Number of enrolled patients (% of total)	Total Number of Centres
USA	110 (20%)	19
Canada	2 (0·4%)	2
Puerto Rico	2 (0·4%)	1
Ireland	2 (0·4%)	1
Italy	48 (8·6%)	2
Portugal	7 (1·3%)	1
Spain	11 (2%)	1
**Turkey**	23 (4·1%)	2
**Argentina**	29 (5·2%)	8
**Bolivia**	2 (0·4%)	8
Chile	39 (7%)	11
**Colombia**	137 (25%)	20
**Costa Rica**	17 (3·1%)	11
**Honduras**	29 (5·2%)	1
**Mexico**	16 (2·9%)	1
**Peru**	83 (15%)	2
Uruguay	0	2
**Panama**	0	1

Total number of centres includes those that had IRB approval and screened but did not have patients included for this analysis. Countries considered low or middle income countries using the World Bank categories are noted in **bold**.

Supplemental Table 2: Comparison of symptoms at presentation, including symptom category and components of each category, between children who survived and those who died.

Symptoms		All	Survived (n=501)	Died (n=56)
Lower Respiratory		262 (47%)	221 (44%)	41 (73%)
	Cough	209 (38%)	176 (35%)	33 (59%)
	Wheeze	63 (11%)	50 (10%)	13 (23%)
	Retractions	164 (29%)	128 (26%)	36 (64%)
Upper Respiratory		165 (30%)	154 (31%)	11 (20%)
	Sore Throat	91 (16%)	87 (17%)	4 (7·1%)
	Rhinorrhoea	95 (17%)	86 (17%)	9 (16%)
	Ear Pain	6 (1%)	6 (1·2%)	0
Systemic		469 (84%)	423 (84%)	46 (82%)
	Fever	428 (77%)	392 (78%)	36 (64%)
	Fatigue	310 (56%)	280 (56%)	30 (54%)
Gastrointestinal		327 (59%)	304 (61%)	23 (41%)
	Abdominal Pain	241 (43%)	226 (45%)	15 (27%)
	Vomiting	238 (43%)	223 (45%)	15 (27%)
	Diarrhoea	187 (34%)	174 (35%)	13 (23%)
Neurologic		214 (38%)	190 (38%)	24 (43%)
	Headache	114 (20%)	108 (22%)	6 (11%)
	Confusion	107 (19%)	87 (17%)	20 (36%)
	Seizure	48 (8·6%)	40 (8%)	8 (14%)
Mucocutaneous		208 (37%)	198 (40%)	10 (18%)
	Rash	147 (26%)	142 (28%)	5 (8·9%)
	Conjunctivitis	118 (21%)	116 (23%)	2 (3·6%)
	Skin Ulcers	8 (1·4%)	7 (1·4%)	1 (1·8%)
	"Strawberry" Tongue/Oral Lesions	64 (12%)	63 (13%)	1 (1·8%)
	Red Hands/Feet	105 (19%)	99 (20%)	6 (11%)
Non-Specific		299 (54%)	263 (52%)	36 (64%)
	Chest Pain	44 (7·9%)	41 (8·2%)	3 (5·4%)
	Myalgias	131 (24%)	124 (25%)	7 (13%)
	Arthralgias	49 (8·8%)	46 (9·2%)	3 (5·4%)
	Shortness of Breath	192 (34%)	160 (32%)	32 (57%)
	Lymphadenopathy, general	53 (9·5%)	50 (10%)	3 (5·4%)
	Lymphadenopathy, cervical	49 (9%)	46 (9·2%)	3 (5·4%)
Bleeding		14 (2·5%)	9 (1·8%)	5 (8·9%)

Data are n (%)

Supplemental Table 3: Association of Comorbidities and Clinical Presentation with Mortality. Odds ratios for mortality calculated using univariate logistic regression

Variable	Mortality with Variable	Mortality without Variable	Odds Ratio	95% CI
**Comorbidities**				
Cardiac	10/43 (23%)	46/514 (9%)	**3·08**	1·43, 6·66
Pulmonary (not asthma)	8/32 (25%)	48/525 (9·1%)	**3·31**	1·41, 7·78
Asthma	5/44 (11%)	51/513 (9·9%)	1·16	0·44, 3·08
Renal	3/22 (14%)	53/535 (9·9%)	1·44	0·41, 5·01
Liver	4/9 (44%)	52/548 (9·5%)	**7·63**	1·99, 29·3
Neurologic	9/58 (16%)	47/499 (9·4%)	1·77	0·82, 3·82
Malignancy	7/20 (35%)	49/537 (9·1%)	**5·36**	2·04, 14·1
Hematologic	5/18 (28%)	51/539 (9·5%)	**3·68**	1·26, 10·7
Obesity	4/74 (5·4%)	52/483 (11%)	0·47	0·17, 1·35
Diabetes	1/11 (9·1%)	55/491 (10%)	0·89	0·11, 7·11
Malnutrition	10/36 (28%)	46/521 (8·8%)	**3·97**	1·80, 8·75
>1 Comorbidity	26/119 (22%)	30/438 (6·9%)	**3·80**	2·15, 6·73
No Comorbidities	15/272 (5·5%)	41/285 (14%)	**0·35**	0·19, 0·64
**Clinical Presentation**				
Known COVID Exposure	23/240 (9·6%)	33/315 (10%)	0·91	0·52, 1·59
Ward Admission Pre-ICU	29/249 (12%)	23/278 (8·3%)	1·46	0·82, 2·60
Fever (>38·3°C) at Admission	6/112 (5·4%)	49/430 (11%)	0·44	0·18, 1·06
Admission O_2 Saturation <94%	21/116 (18%)	34/417 (8·2%)	**2·49**	1·38, 4·49
Dehydration at Admission	10/87 (11%)	46/468 (9·8%)	1·19	0·58, 2·46
Delayed Admission Capillary Refill	25/141 (18%)	18/229 (7·9%)	**2·53**	1·32, 4·82
**Presenting Symptoms**				
Lower Respiratory	41/262 (16%)	15/295 (5·1%)	**3·46**	1·87, 6·42
Upper Respiratory	11/165 (6·7%)	45/392 (11%)	0·55	0·28, 1·09
Systemic	46/469 (9·8%)	10/88 (11%)	0·85	0·41, 1·75
Gastrointestinal	23/327 (7%)	33/230 (14%)	**0·45**	0·26, 0·79
Neurologic	24/214 (11%)	32/343 (9·3%)	1·23	0·70, 2·15
Mucocutaneous	10/208 (4·8%)	46/349 (13%)	**0·33**	0·16, 0·67
Non-Specific	36/299 (12%)	20/258 (7·8%)	1·63	0·92, 2·89

Statistically significant results in **bold.**

Supplemental Table 4: Laboratory abnormalities identified on hospital day 1 significantly* associated with mortality. Percentage calculated based on entire cohort.

	**All** (n=557)	**Survived** (n=501)	**Died** (n=56)
pH	7·36 (7·29, 7·40)	7·36 (7·30, 7·41)	7·31 (7·21, 7·39)
BUN (mg/dL)	16 (9, 30)	15 (9, 28)	19·5 (10, 39)
Sodium (mEq/L)	136 (133, 139)	136 (133, 139)	138 (135, 142)
Lactate (mmol/L)	2 (1·3, 3·3)	1·9 (1·2, 3·2)	2·5 (1·7, 4·6)
Procalcitonin > 2 ng/mL	65 (12%)	63 (12·6%)	2 (3·6%)
C-Reactive Protein (4 mg/dL)	10 (2·3, 20·1)	10 (2·4, 20·5)	4·9 (0·9, 10)
Fibrinogen (mg/dL)	450 (274, 639)	465 (279, 645)	248 (168, 446)
BNP >60 pg/mL	129 (23%)	122 (24%)	7 (13%)

Data are median (IQR) or n (%)

* $p<0.05$ using Wilcoxon rank-sum or chi-square test

Abbreviations: BUN = blood urea nitrogen; BNP = brain natriuretic peptide

SI Conversion Factors: To convert BUN, multiply by 0.357. To convert C-reactive protein, multiply by 10

Supplemental Table 5 Complete list of hospital diagnoses and complications including odds ratio for mortality of each diagnosis in univariate logistic regression analysis.

Diagnosis/Complication	Prevalence	Mortality With Diagnosis	Mortality Without Diagnosis	Odds Ratio	95% CI
Viral Pneumonia/Pneumonitis	183 (33%)	27/183 (15%)	29/373 (7·8%)	**2·05**	1·18, 3·58
Bacterial Pneumonia	113 (20%)	22/113 (19%)	34/443 (7·7%)	**2·91**	1·62, 5·21
ARDS	157 (28%)	38/157 (24%)	18/400 (4·5%)	**6·78**	3·73, 12·3
Pneumothorax	15 (2·7%)	10/15 (67%)	46/541 (8·5%)	**21·5**	7·06, 65·6
Pleural Effusion	79 (14%)	10/79 (13%)	46/478 (9·6%)	1·36	0·66, 2·82
Bronchiolitis	26 (4·7%)	2/26 (7·7%)	54/531 (10%)	0·74	0·17, 3·20
Meningitis	29 (5·2%)	5/29 (17%)	51/528 (9·7%)	1·95	0·72, 5·33
Seizure	53 (9·5%)	12/53 (23%)	44/504 (8·7%)	**3·06**	1·50, 6·25
Stroke	19 (3·4%)	8/19 (42%)	48/491 (8·9%)	**7·42**	2·85, 19·3
Heart Failure	86 (15%)	23/86 (27%)	33/471 (7%)	**4·85**	2·67, 8·78
Myocarditis/Cardiomyopathy	101 (18%)	8/101 (7·9%)	48/456 (11%)	0·73	0·33, 1·60
Arrhythmia	39 (7%)	10/39 (26%)	46/518 (8·9%)	**3·54**	1·62, 7·72
Cardiac Arrest	56 (10%)	47/56 (84%)	9/501 (1·8%)	**285**	108, 754
Bacteraemia	68 (12%)	22/68 (32%)	33/488 (6·8%)	**6·59**	3·55, 12·2
Coagulopathy	98 (18%)	21/98 (21%)	35/459 (7·6%)	**3·30**	1·83, 5·98
Anaemia	198 (36%)	34/198 (17%)	22/359 (6·1%)	**3·18**	1·80, 5·60
Rhabdomyolysis	6 (1·1%)	0/6 (0%)	56/550 (10%)	-	-
Acute Kidney Injury	88 (16%)	24/88 (27%)	32/469 (6·8%)	**5·12**	2·84, 9·24
Gastroenteritis	11 (2%)	4/11 (36%)	52/546 (9·5%)	**5·43**	1·54, 19·2
Pancreatitis	13 (2·3%)	3/13 (23%)	53/543 (9·8%)	2·77	0·74, 10·4
Acute Hepatic Injury	51 (9·2%)	11/51 (22%)	45/506 (8·9%)	**2·82**	1·35, 5·87
MIS-C	188 (34%)	6/188 (3·2%)	50/368 (14%)	**0·21**	0·09, 0·50

Data are n (%). Statistically significant results in **bold.**
Abbreviations: ARDS = acute respiratory distress syndrome; MIS-C = multisystem inflammatory syndrome in children

Supplemental Table 6. Mortality rates for factors significantly associated with mortality in adjusted logistic regression stratified by age.

	<2 years old		≥2 years old	
	**Mortality**		**Mortality**	
**Variable**	With Variable	Without Variable	With Variable	Without Variable
Admission O_2 Saturation <94%	8/32 (25%)	12/97 (12%)	13/84 (15%)	22/320 (6.9%)
Pulmonary Comorbidity	2/9 (22%)	18/125 (14%)	6//23 (26%)	30/400 (7.5%)
Malnutrition	5/15 (33%)	15/119 (13%)	5/21 (24%)	31/402 (7.7%)
Lower Respiratory Symptoms	17/75 (23%)	3/59 (5.1%)	24/187 (13%)	12/236 (5.1%)
Gastrointestinal Symptoms	5/37 (14%)	15/97 (15%)	18/290 (6.2%)	18/133 (14%)
Mucocutaneous Symptoms	2/21 (9.5%)	18/113 (16%)	8/187 (4.3%)	28/236 (12%)
Day 1 Invasive Ventilation	11/40 (28%)	9/89 (10%)	17/94 (18%)	19/323 (5.9%)
Prophylactic Anticoagulation	3/21 (14%)	17/113 (15%)	8/174 (4.6%)	28/249 (11%)
IVIG	6/37 (16%)	14/97 (14%)	7/225 (3.1%)	29/198 (15%)
Dexamethasone	5/35 (14%)	15/99 (15%)	12/122 (9.8%)	24/301 (7.8%)
Methylprednisolone	5/28 (18%)	15/106 (14%)	7/192 (3.7%)	29/231 (13%)
Viral Pneumonia	15/60 (25%)	5/74 (6.8%)	12/123 (9.8%)	24/299 (8%)
ARDS	14/42 (33%)	6/92 (6.5%)	24/115 (21%)	12/308 (3.9%)
Bacteraemia	10/28 (36%)	9/105 (8.6%)	12/40 (30%)	24/383 (6.3%)
Acute Kidney Injury	8/13 (62%)	12/121 (10%)	16/75 (21%)	20/348 (5.8%)
Acute Liver Injury	4/6 (67%)	16/128 (13%)	7/45 (16%)	29/378 (7.7%)
MIS-C	3/20 (15%)	17/114 (15%)	3/168 (1.8%)	33/254 (13%)

Supplemental Figure 1. Hospital mortality rate stratified by age

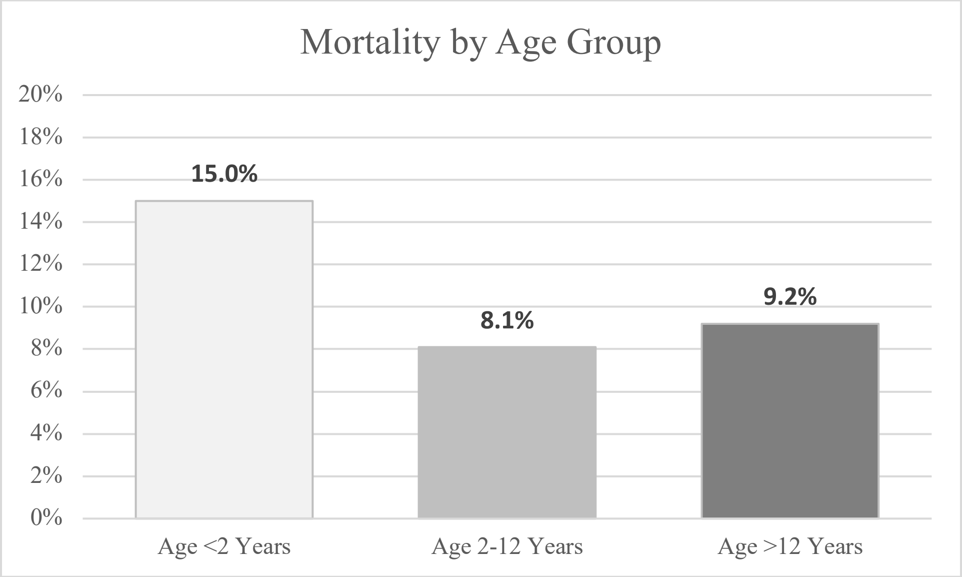

Supplemental Figure 2: Hospital mortality rate stratified by age and a.) **MIS-C or** b.) **No MIS-C**

a.)

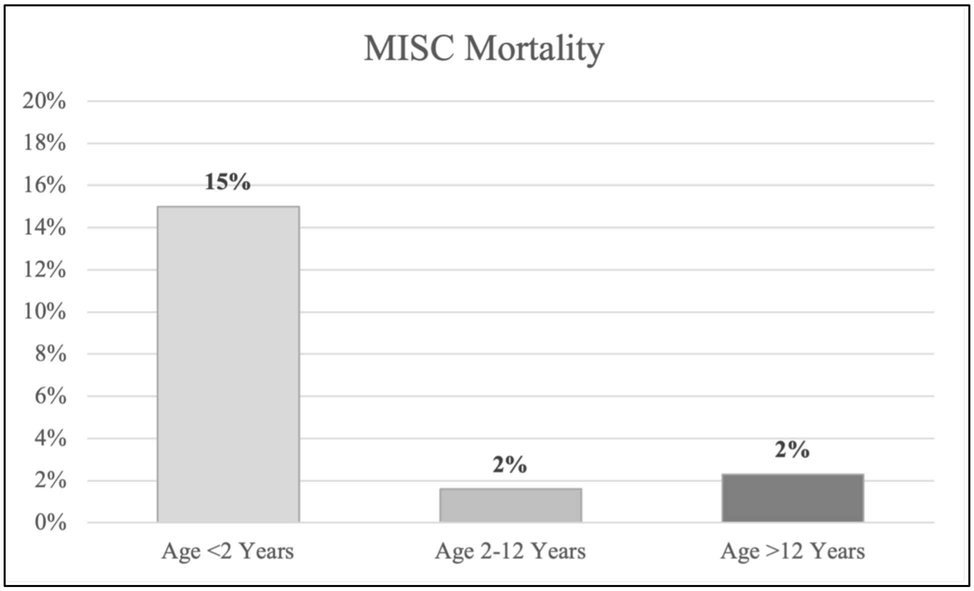

b.)

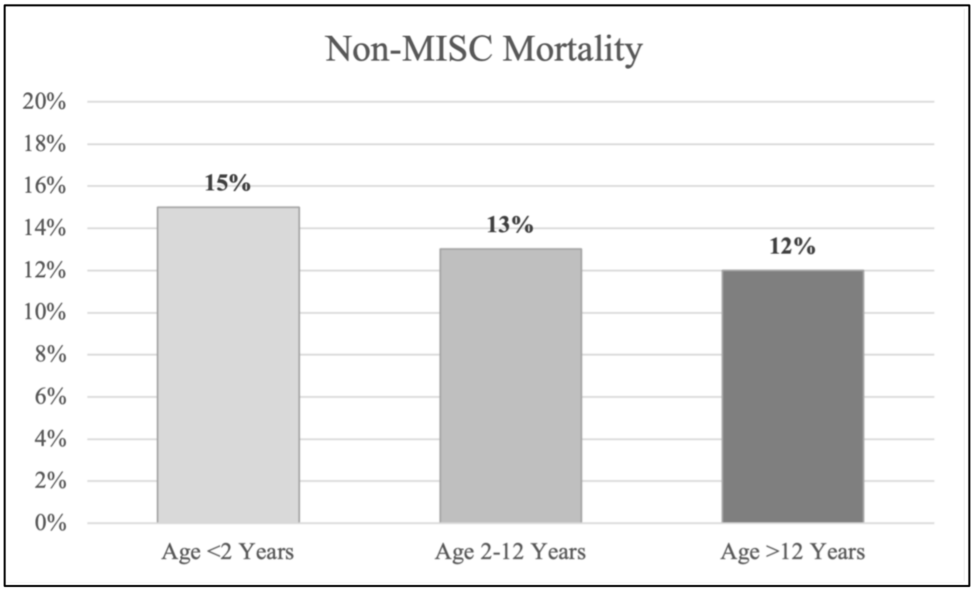

Supplement: Supplementary file 2 [file mmc2.pdf]
