# Supplementary material for: Paediatric critical COVID-19 and mortality in a multinational prospective cohort
Source: Lancet Reg Health Am. 2022 May 17;12:100272. doi: 10.1016/j.lana.2022.100272 (PMC9111167; doi:10.1016/j.lana.2022.100272)
Supplement: Supplementary file 3 [file mmc3.docx]

***Editorial disclaimer:*** *This translation in Spanish was submitted by the authors and we reproduce it as supplied. It has not been peer reviewed. Our editorial processes have only been applied to the original abstract in English, which should serve as reference for this manuscript.*

**Resumen**

**Antecedentes**: Comprender la enfermedad crítica pediátrica por coronavirus 2019 (COVID-19) y evaluar los factores asociados con la mortalidad en niños de países de ingresos altos y medios-bajos.

**Métodos**: Estudio observacional prospectivo de niños gravemente enfermos hospitalizados por COVID-19 en 18 países de América del Norte, América Latina y Europa entre el 1 de abril y el 31 de diciembre de 2020. Las asociaciones con la mortalidad se evaluaron mediante regresión logística.

**Resultados**: 557 pacientes (mediana de edad, 8 años; 24% <2 años) se reclutaron en 55 sitios (63% latinoamericanos). Las modalidades de soporte más comunes fueron la ventilación invasiva (41%) o no invasiva (20%) y los vasopresores (56%) fueron. La mortalidad hospitalaria fue del 10% y más alta en niños <2 años (15%; odds ratio 1,94; IC del 95%: 1,08-3,49). La mayoría de los que murieron tenían enfermedad pulmonar. Cuando se ajustaron por edad, sexo, región y gravedad de la enfermedad, los factores asociados a la mortalidad incluyeron comorbilidades cardíacas (aOR 2·89; IC del 95%: 1,2-6,94) o pulmonares (aOR 4,43; IC del 95%: 1,70-11,5), hipoxemia a la admisión (aOR 2,44; IC del 95%: 1,30-4,57) y síntomas respiratorios bajos (aOR 2,96; IC del 95%: 1,57-5,59). Tener MIS-C (aOR 0,25; IC del 95%: 0,1-0,61) y recibir metilprednisolona (aOR 0,5; IC del 95%: 0,25-0,99), IGIV (aOR 0,32; IC del 95%: 0,16-0,62) o anticoagulación (aOR 0,49; IC del 95%: 0,25-0,95) se asociaron con una menor mortalidad, aunque estas asociaciones se limitan a niños de >2 años.

**Interpretación**: Identificamos factores asociados con la mortalidad por COVID-19 en niños gravemente enfermos tanto de países de ingresos altos y medio bajos, incluida una mayor mortalidad a una edad más temprana y relacionada a enfermedad pulmonar por COVID, pero una menor mortalidad en MIS-C. Se necesitan estudios de investigación adicionales sobre los tratamientos óptimos para los niños más pequeños y para la insuficiencia respiratoria en la COVID-19 pediátrica.

**Financiación:** Este estudio no recibió financiación.
